# Supplementary material for: Plant conversions and abatement technologies cannot prevent stranding of power plant assets in 2 °C scenarios
Source: Nat Commun. 2022 Feb 10;13:806. doi: 10.1038/s41467-022-28458-7 (PMC8831661; doi:10.1038/s41467-022-28458-7)
Supplement: Supplementary file 1 — Supplementary Information pdf [file 41467_2022_28458_MOESM1_ESM.pdf]

Supplementary Information

Plant conversions and abatement technologies cannot prevent stranding of power plant assets in 2°C scenarios

Yangsiyu Lu, Francois Cohen, Stephen M Smith, Alexander Pfeiffer

Supplementary Table 1: Overview of IAM models used in this study 2

Supplementary Table 2: Assumed utilisation rates 5

Supplementary Table 3: Overview of power generators in our dataset 6

Supplementary Table 4: Comparison of lifetime assumptions 7

Supplementary Figure 1: Breakdown of committed electricity generation between 2021 and 2100 8

Supplementary Figure 2: Global electricity generation from different technologies between 2021 and 2100 in AMPERE scenarios 9

Supplementary Figure 3: Estimated stranded generation in all technologies deployed scenarios (2021-2100) by type of fuel 10

Supplementary Figure 4: Sensitivity of (a) estimated electricity generation and (b) stranded generation to utilisation rates and asset lifetime. 11

Supplementary Figure 5: Estimated stranded generation using the IPCC SR1.5 database, for (a) 1.5 degrees and (b) 2 degrees scenarios 13

Supplementary Figure 6: CO_2 capture from biomass and other sources in the AMPERE all technology deployed scenarios and the IEA Sustainable Development scenarios 14

Supplementary Figure 7: (a) Historical and (b) future utilisation rates in IEA World Energy Outlook 15

Supplementary Information References 16

Overview of IAM models used in this study. Supplementary Table 1 presents the general characteristics (panel a), socioeconomic drivers (panel b) and key energy technology assumptions (panel c) of these Integrated Assessment Models (IAMs). The information is compiled from IPCC (2014) and Kriegler *et al.*(2015). Supplementary Table 1, panel c, shows that plant conversions from coal to gas or coal to biomass are not included in the calculations of electricity generation by fuels in the IAMs, except for GCAM. Probably, this implies that GCAM might allow for more coal at the beginning of the century, since generation can be converted into gas or biomass. However, it is difficult to determine the separate impact of this assumption, since other assumptions in the models may also create differences in electricity generation levels by fuels in the IAMs. Due to the heterogeneity of assumptions on technology development, it is essential to rely on a set of different IAMs to obtain robust results.

Supplementary Table 1: Overview of IAM models used in this study

a) General characteristics

Model name	Institution	Reference	Equilibrium type	Modelling approach	Time horizon
GCAM	JGCRI, US	JGCRI (2021)	Partial	Recursive dynamic	2100
IMACLIM	CIRED and SMASH,	Sassi et al. (2010)	General	Recursive dynamic	2100
IMAGE	UU and PBL, Netherlands	Bouwman et al. (2006)	Partial	Recursive dynamic	2100
MESSAGE	IIASA, Austria	Messner et al. (2000)	General	Intertemporal optimization	2100
POLES	EC-JRC, Belgium	IPTS (2010)	Partial	Recursive dynamic	2100
REMIND	PIK, Germany	Leimbach et al. (2010)	General	Intertemporal optimization	2100
WITCH	FEEM and CMCC, Italy	De Cian et al., (2009)	General	Intertemporal optimization	2100

b) Social economic drivers

Model name	Exogenous drivers	Endogenous drivers
GCAM	Population, GDP, labour participation rate, labour productivity	None
IMACLIM	Labour productivity, energy technical progress, population, active population	None
IMAGE	Exogenous GDP, GDP per capita, population	Energy demand, renewable price, fossil fuel prices, carbon prices, technology progress, energy intensity, preferences, learning by doing, agricultural demand, value added
MESSAGE	Labour productivity, energy technical progress, GDP per capita, population	None
POLES	Exogenous GDP, population	Value added, mobility needs, fossil fuel prices, buildings surfaces
REMIND	Labour productivity, energy efficiency parameters of the production function, population	Investments in industrial capital stock and specific energy technology capital stocks, endogenous learning-by-doing for wind and solar power as well as electric and fuel cell vehicle technologies (global learning curve, internalized spillovers).
WITCH	Total factor productivity, labour productivity, capital technical progress	None

c) Key energy technology assumptions

Model name	Energy Conversion technologies	Energy technology choice, substitutability and deployment
GCAM	CHP, hydrogen from coal, oil, gas, and biomass, w/o and w/ CCS, nuclear and solar thermochemical, fuel to gas, coal	Logit choice model with usually high substitutability

	to gas w/o CCS, biomass (w/o and w/ CCS), fuel to liquid, coal to liquids (w/o and w/ CCS), gas to liquids (w/o and w/ CCS), biomass to liquids (w/o and w/ CCS)	
IMACLIM	Fuel to liquid	Logit choice model, mostly high substitutability in some sectors and mostly low substitutability in other sectors, expansion and decline constraints, system integration constraints
IMAGE	CHP, hydrogen	Logit choice model, mostly high substitutability, expansion and decline constraints, system integration constraints
MESSAGE	CHP, hydrogen, fuel to gas, fuel to liquid	Linear choice (lowest cost), mostly high substitutability, expansion and decline constraints, system integration constraints
POLES	CHP, hydrogen, fuel to liquid	Logit choice model, mostly high substitutability, expansion and decline constraints
REMIND	CHP, Heat pumps, hydrogen (from fossil fuels and biomass with and w/o CCS; electrolytic hydrogen), fuel to gas, fuel to liquid (from fossil fuels and biomass with and w/o CCS), heat plants	Discrete technology choices with high to full substitutability, expansion and decline constraints, system integration constraints
WITCH	None	No discrete technology choices, mostly high substitutability, expansion and decline constraints, system integration constraints

Supplementary Table 2: Assumed utilisation rates

	Baseline	Minimum	Maximum
Coal	56%	54%	65%
Gas	39%	37%	40%
Oil	23%	21%	31%

The baseline represents the average value of utilisation rates in the Stated Policies Scenarios from IEA World Energy Outlook 2019. The maximum and minimum cases use the maximum and minimum historical utilisation rates in the years 2004-2018 respectively, extracted from IEA World Energy Outlook 2005-2019.

Supplementary Table 3: Overview of power generators in our dataset

	Total capacity (GW)	Average online year	Number of generator units
Panel A: In operation in 2019			
Coal	2217	1994	9924
Gas	1495	1997	28423
Oil	436	1992	29296
Bio gas	19	2000	2477
Biomass	36	2004	4082
Bio oil	10	1994	419
Geothermal	15	2000	642
Nuclear	414	1989	444
Solar	128	2012	15581
Waste	339	2003	6759
Hydro	1229	1981	51633
Unknown	11	1982	1088
All	6349		
Panel B: In the pipeline in 2019			
Coal	959	2022	2094
Gas	682	2022	2840
Oil	42	2022	696
Bio gas	4	2022	213
Biomass	13	2022	582
Bio oil	1	2022	25
Geothermal	11	2022	290
Nuclear	295	2023	268
Solar	159	2022	2711
Waste	87	2022	971
Hydro	688	2022	9066
Unknown	0.4	2023	15
All	2941		

Supplementary Table 4: Comparison of lifetime assumptions (Years)

Fuel	Average lifetime assumed in our baseline	Average lifetime in Tong *et al.* (2019)	Technical lifetime in IRENA (2017)	Economic lifetime in IRENA (2017)
Coal	40	36	50	25
Gas	39	37	30	15
Oil	39	34	50	25

Separate pathways of electricity generation by fuel. Supplementary Figure 1 shows the committed electricity generation between 2021 and 2100 by fuel. It displays the electricity generation forecast in the all technologies deployed scenario modelled by MESSAGE model for a climate stabilisation at 450 ppm. In this example, we observe that coal and oil assets are at high risk of stranding, while gas assets are at lower risk of stranding. Please note that this is only one example for one IAM and one scenario. Results from different models and scenarios would differ significantly.

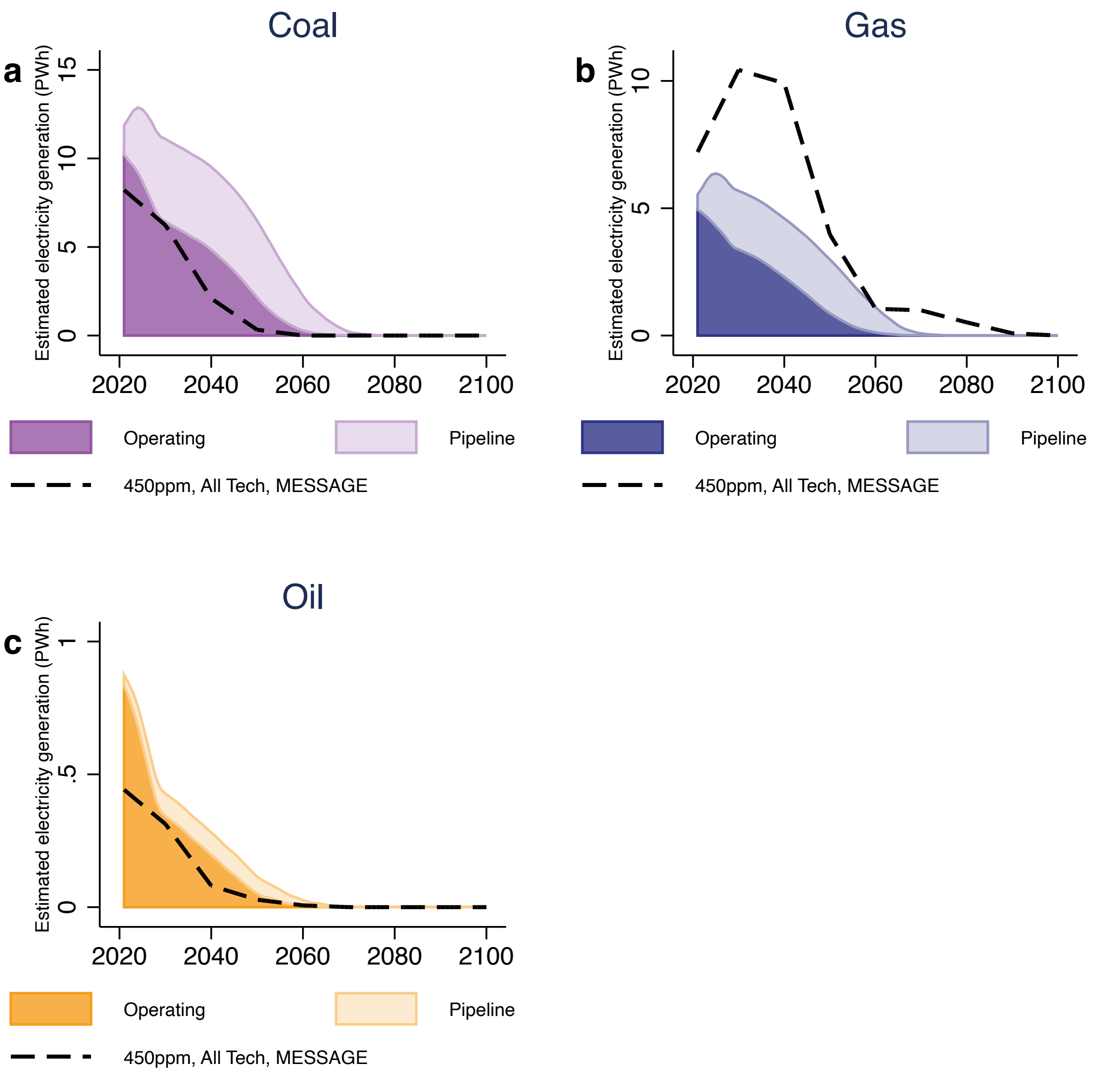

Supplementary Figure 1: Breakdown of committed electricity generation between 2021 and 2100 for (a) coal, (b) gas and (c) oil. Estimates of the amount of future electricity that can be generated from currently operating and in-the-pipeline power plants by fuel. Darker shading indicates the expected generation from currently operating plants, while lighter shading indicates the expected generation from in-the-pipeline plants. The black dashed line is an example for the electricity that could be produced based on one scenario from one Integrated Assessment Model. The specific example is the 450ppm, all technologies deployed scenario obtained from the MESSAGE model.

Global electricity generation by technology in the all technologies deployed scenarios. Supplementary Figure 2 shows the global electricity generation by technology in each IAM.

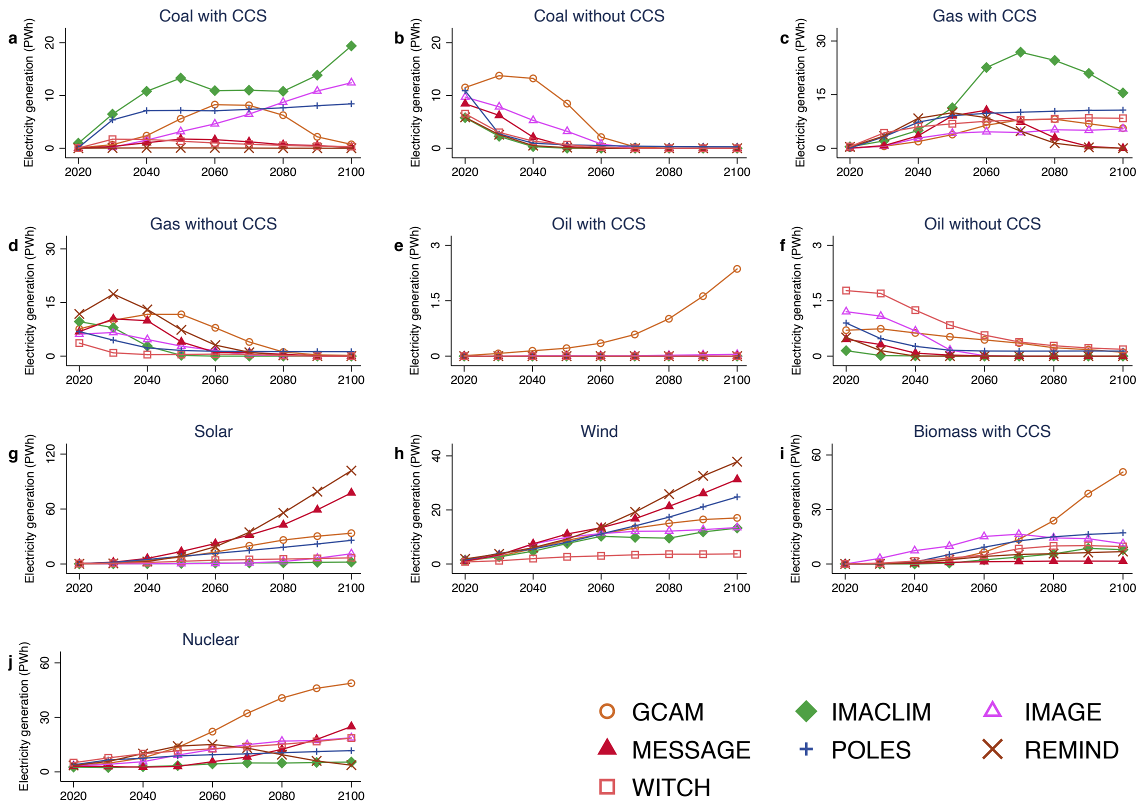

Supplementary Figure 2: Global electricity generation from different technologies between 2021 and 2100 in AMPERE scenarios (all technology deployed, 450ppm optimal scenarios). Electricity generation from (a) coal plants with carbon capture and storage (CCS), (b) coal plants without CCS, (c) gas plants with CCS, (d) gas plants without CCS, (e) oil plants with CCS, (f) oil plants without CCS, (g) solar plants, (h) wind plants, (i) biomass plants with CCS, and (j) nuclear plants.

Estimates of stranded generation by fuel. Figure 2 presented the amount of stranded generation for all types of fossil fuel power plants (including coal-, gas-, and oil-fired). In Supplementary Figure 3, we show the breakdown of stranded generation by fuel, for: a) all fuels (same as Figure 2); b) coal; c) gas; and d) oil. Coal-fired power plants make most of stranded generation (82 percent in the across all IAMs), followed by gas-fired power plants contributing 16 percent in the across model mean. Due to their limited capacity, oil-fired plants only constitute a very small share of stranded generation.

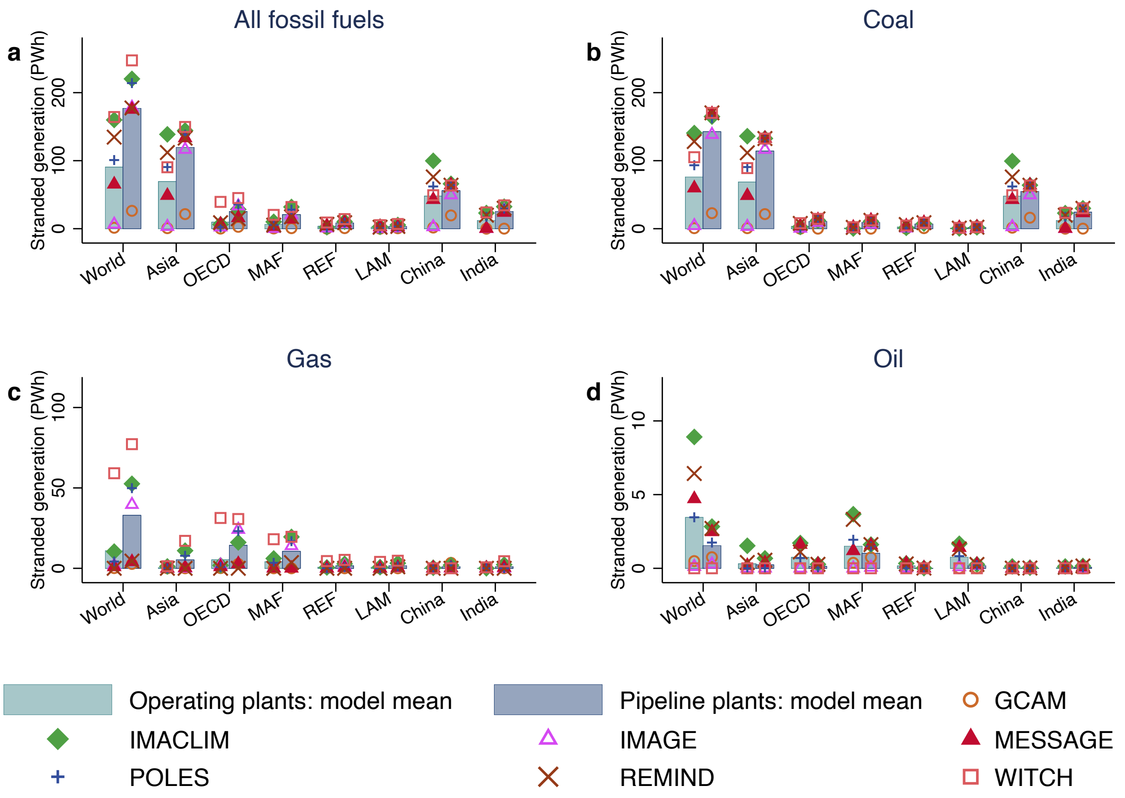

Supplementary Figure 3: Estimated stranded generation in all technologies deployed scenarios (2021-2100) by type of fuel: (a) all fuels; (b) coal; (c) gas; and d) oil. Estimated amount of stranded generation of fossil fuel power plants from 2021 to 2100 in the all technologies deployed scenarios of AMPERE consistent with greenhouse gas concentrations to stabilise at 450ppm. We assume no conversion of any existing fossil fuel fired power plants. Scatter points represent the estimation from individual Integrated Assessment Model and bars show the means across all models. MAF, LAM and REF refer to the Middle East and Africa, Latin America and Reforming Economies, respectively.

Sensitivity analysis for lifetimes and utilisation rates. In Supplementary Figure 4, we check the sensitivity of the estimated electricity generation and the amount of stranded generation to the choice of utilisation rates and lifetimes. For utilisation rates, the baseline uses the average fuel-specific utilisation rates of the Stated Policy Scenarios (2018-2040) from IEA World Energy Outlook (2019) (IEA 2019). The maximum and minimum cases rely on the maximum and minimum fuel-specific utilisation rates observed during years 2004-2018, extracted from IEA World Energy Outlook 2005-2019. For lifetime assumptions, baseline assumes that power generators will operate until the end of their estimated expected lifetime. The reduce and extend cases assume a decrease / increase in lifetime by 10 percent compared to the baseline.

Supplementary Figure 4 shows that higher utilisation rates and longer lifetime assumptions lead to more potential fossil fuel electricity generation in the future and therefore more stranded generation. However, even though the share of stranded generation increases with longer lifetimes and higher utilisation rates, it remains close to about 50 percent of future electricity generation in most cases.

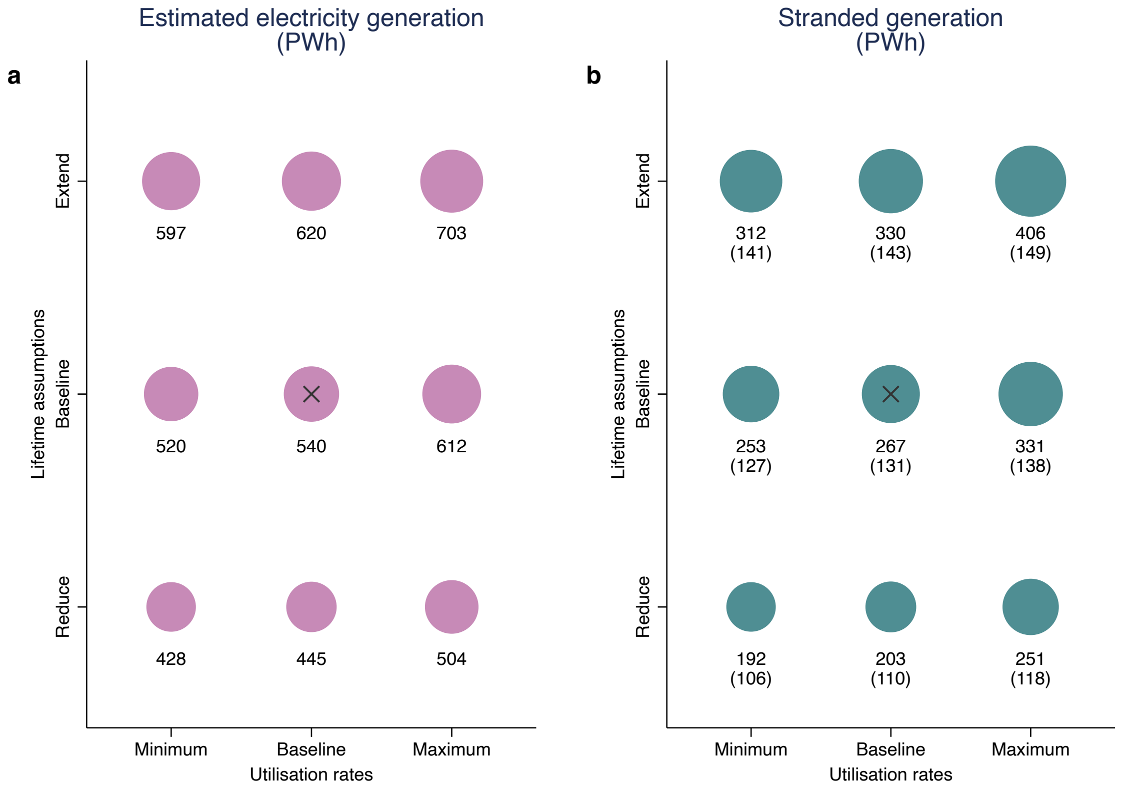

Supplementary Figure 4: Sensitivity of (a) estimated electricity generation and (b) stranded generation to utilisation rates and asset lifetime. All cases show the total estimated electricity generation and stranded generation of operating and in-the-pipeline fossil fuel power plants (coal-, gas- and oil-fired) from 2021 to 2100 in the all

technologies deployed scenarios, without any conversion. The circles for panel (b) provide the mean across 7 Integrated Assessment Models (GCAM, IMACLIM, IMAGE, MESSAGE, POLES, REMIND, and WITCH). The standard deviation reported in parentheses in panel (b) accounts for the variation across models. The baseline assumption based results presented in the main results are emphasised with the x symbol.

Estimates of stranded generation using the IPCC SR1.5 database. Our results are obtained by using AMPERE scenarios. Other databases of IAMs could be used to calculate stranded generation with our methodology. We show the results of stranded generation using IPCC SR1.5 database in Supplementary Figure 5. The average amount of global stranded generation in 2°C scenarios of IPCC SR1.5 database is about 300 PWh, which is 12 percent higher than the amount in AMPERE 2°C scenarios of Figure 2. This is mostly because Figure 2 assumes that all technologies reducing the level of stranded generation are fully deployed, while 2°C scenarios of IPCC SR1.5 have varying assumptions on technology development. The amount of global stranded generation under 1.5°C scenarios is higher by about 17 percent compared to 2°C scenarios in IPCC SR1.5 databases. Besides, the order of magnitude, regional distribution and model features of stranded generation are very similar. For example, the WITCH model shows the highest amount of stranded generation, while GCAM model has the lowest amount.

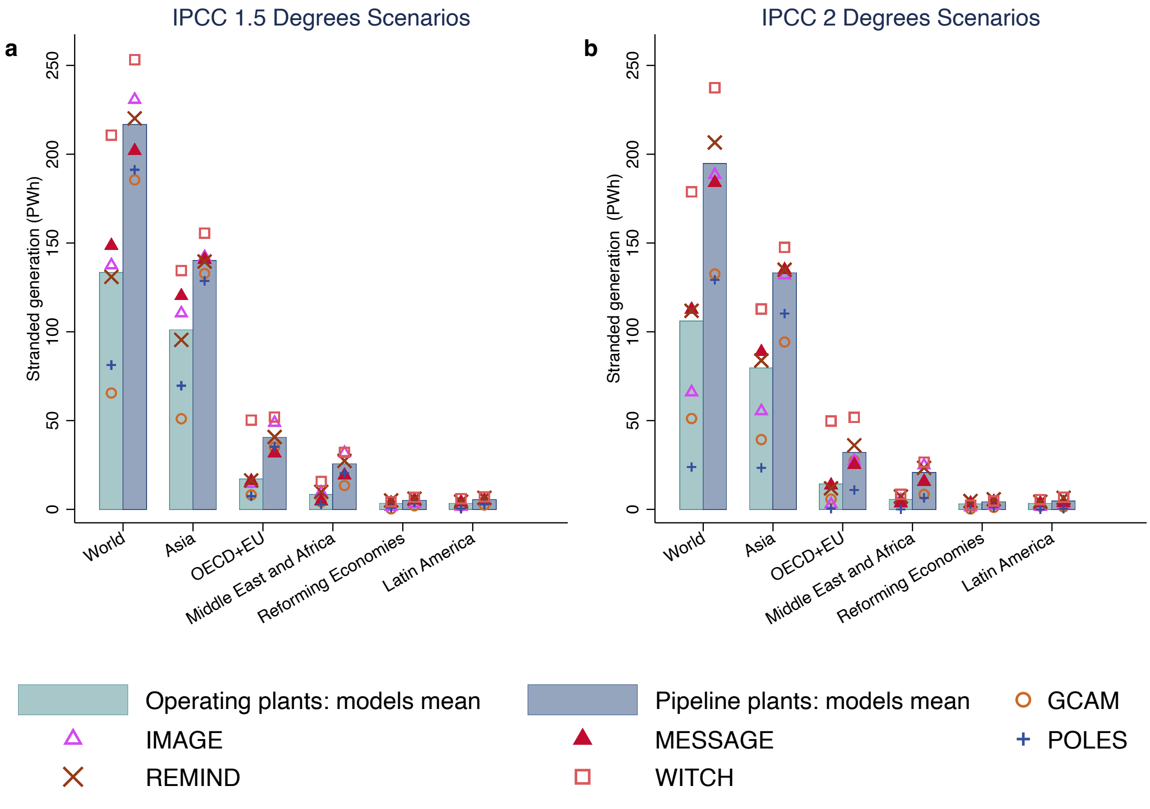

Supplementary Figure 5: Estimated stranded generation using the IPCC SR1.5 database, for (a) 1.5 degrees and (b) 2 degrees scenarios (2021-2100). Scatter points represent the mean value of estimations from individual IAMs and bars show the mean value across all models.

Comparison of CO_2 capture. We compare the CO_2 captured in the AMPERE all technology deployed scenarios with the IEA's Sustainable Development Scenario (IEA 2020) in Supplementary Figure 6. The AMPERE scenarios and the IEA's Sustainable Development Scenario make relatively similar assumptions for CCS take-up from all sources except biomass. However, assumptions regarding biomass with CCS are very different. The IEA's Sustainable Development Scenario assumes a late take-up of biomass with CCS, while in the all technology deployed scenarios of AMPERE, this technology is being deployed at scale. In the section on Impact of energy demand, alternative electricity sources, CCS and bioenergy availability, we discuss the possible impact of low technology diffusion on the amount of stranded generation.

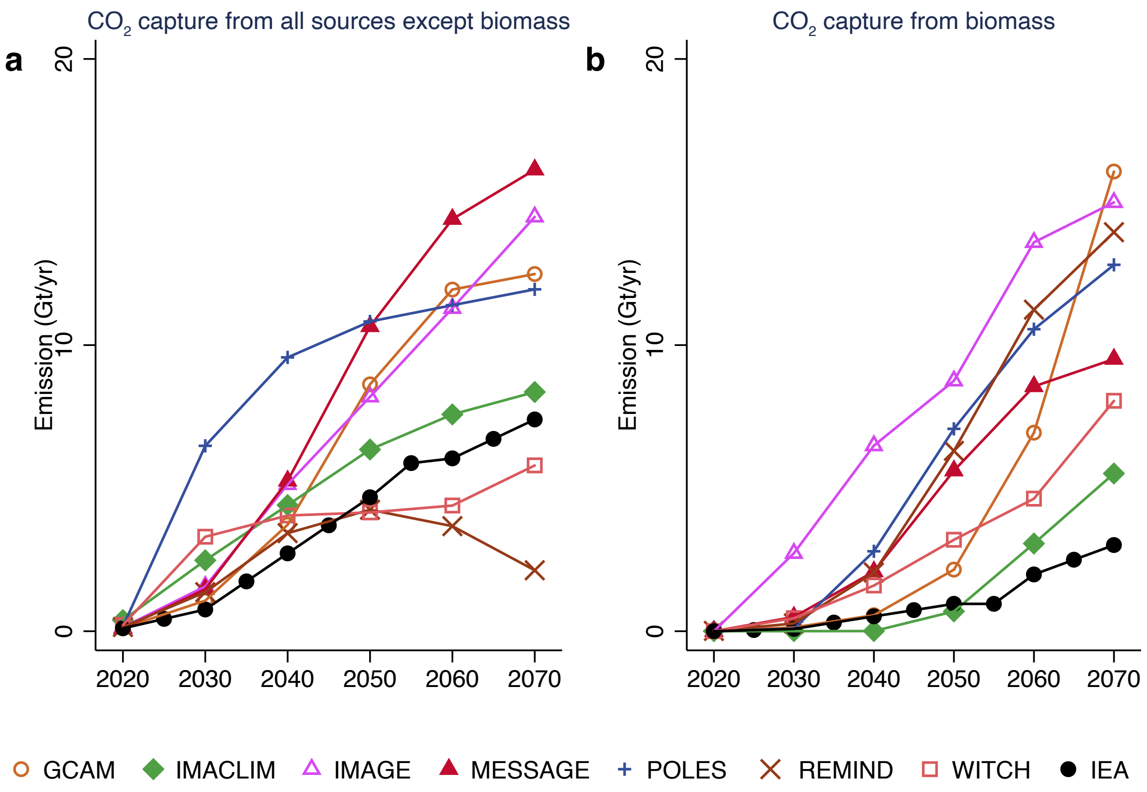

Supplementary Figure 6: CO_2 capture from biomass and other sources in the AMPERE all technology deployed scenarios and the IEA Sustainable Development scenarios (2021-2070). We provide the information separately for (a) carbon capture and storage (CCS) from all sources except biomass and (b) CCS from biomass until 2070. The IEA scenarios only model pathways until 2070.

Utilisation rates. We extract global fuel-specific load factors (i.e. utilisation rate) for power generators from IEA's World Energy Outlook and present them in Supplementary Figure 7.

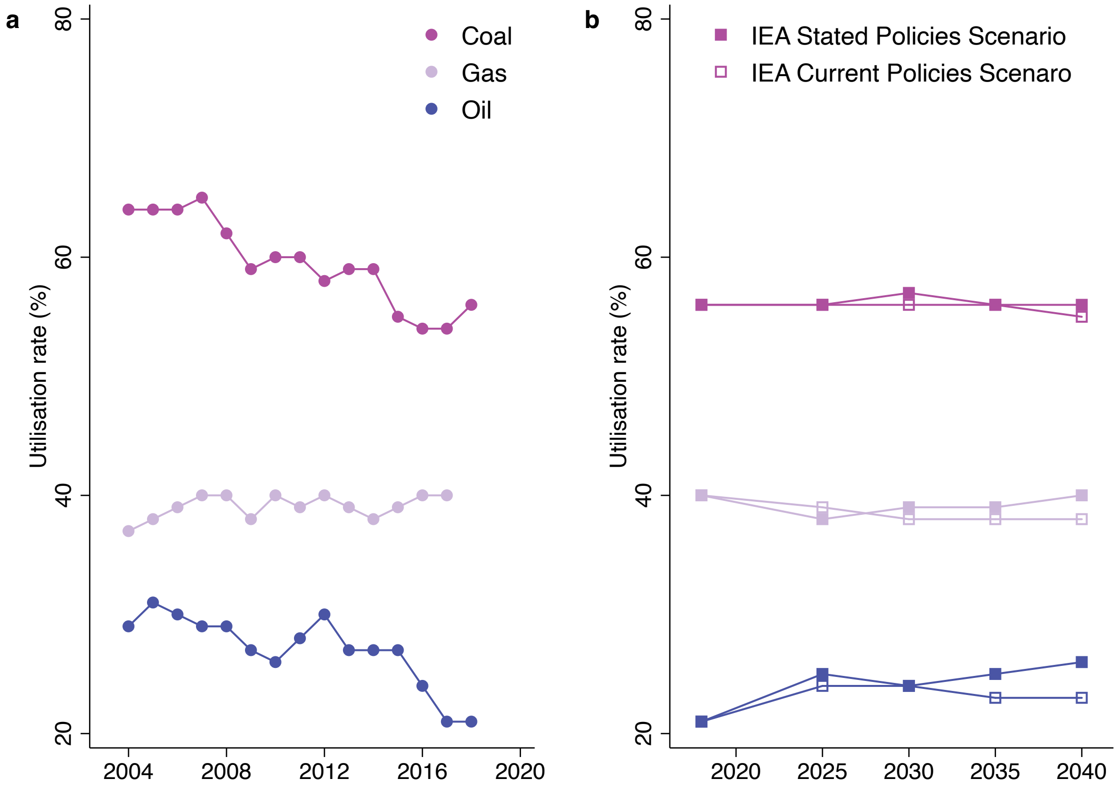

Supplementary Figure 7: (a) Historical and (b) future utilisation rates in IEA World Energy Outlook. Utilisation rates in IEA Stated Policies Scenario and Current Policies Scenario are extracted from IEA's World Energy Outlook 2019. Historical utilization rates are extracted from IEA World Energy Outlook 2005-2019.

Supplementary Information References

Bouwman, A F, T Kram, and K Klein Goldewijk. 2006. Integrated Modelling of Global Environmental Change. *An Overview of IMAGE* 2 (4): 225–28.

Cian, Enrica De, Valentina Bosetti, Alessandra Sgobbi, and Massimo Tavoni. 2009. The 2008 WITCH Model: New Model Features and Baseline.

IEA. 2019. World Energy Outlook. Paris. https://www.iea.org/reports/world-energy-outlook-2019.

———. 2020. CCUS in Clean Energy Transitions. Paris, France. https://www.iea.org/reports/ccus-in-clean-energy-transitions.

IPCC. 2014. Climate Change 2014: Mitigation of Climate Change. Contribution of Working Group III to the Fifth Assessment Report of the Intergovernmental Panel on Climate Change. Cambridge, United Kingdom.

IPTS. 2010. Prospective Outlook on Long-Term Energy Systems. POLES Manual, Version 6.1.

IRENA. 2017. Stranded Assets and Renewables: How the Energy Transition Affects the Value of Energy Reserves, Buildings and Capital Stock. Abu Dhabi. www.irena.org/remap.

JGCRI. 2021. GCAM Online Documentation. *Global Change Analysis Model*.

Kriegler, Elmar, Nils Petermann, Volker Krey, Valeria Jana Schwanitz, Gunnar Luderer, Shuichi Ashina, Valentina Bosetti, et al. 2015. Diagnostic Indicators for Integrated Assessment Models of Climate Policy. *Technological Forecasting and Social Change* 90 (PA): 45–61. https://doi.org/10.1016/j.techfore.2013.09.020.

Leimbach, Marian, Nico Bauer, Lavinia Baumstark, Michael Luken, and Ottmar Edenhofer. 2010. Technological Change and International Trade-Insights from REMIND-R. *The Energy Journal* 31 (Special Issue).

Messner, Sabine, and Leo Schrattenholzer. 2000. MESSAGE–MACRO: Linking an Energy Supply Model with a Macroeconomic Module and Solving It Iteratively. *Energy* 25 (3): 267–82.

Sassi, Olivier, Renaud Crassous, Jean-Charles Hourcade, Vincent Gitz, Henri Waisman, and Céline Guivarch. 2010. IMACLIM-R: A Modelling Framework to Simulate Sustainable Development Pathways. *International Journal of Global Environmental Issues* 10 (1–2): 5–24.

Tong, Dan, Qiang Zhang, Yixuan Zheng, Ken Caldeira, Christine Shearer, Chaopeng Hong, Yue Qin, and Steven J. Davis. 2019. Committed Emissions from Existing Energy Infrastructure Jeopardize 1.5 °C Climate Target. *Nature* 572 (7769): 373–77. https://doi.org/10.1038/s41586-019-1364-3.
